# The Activity of CCL18 is Principally Mediated through Interaction with Glycosaminoglycans

**DOI:** 10.3389/fimmu.2013.00193

**Published:** 2013-07-15

**Authors:** Sonja Krohn, Alexandre Garin, Cem Gabay, Amanda E. I. Proudfoot

**Affiliations:** ^1^Department of Immunology, Merck Serono Geneva Research Centre, Geneva, Switzerland; ^2^Division of Rheumatology, University Hospitals of Geneva, Geneva, Switzerland; ^3^Department of Pathology-Immunology, University of Geneva School of Medicine, Geneva, Switzerland

**Keywords:** chemokines, CCL18, synovial fluid, glycosaminoglycan, BBXB motif, Evasin

## Abstract

The CC chemokine ligand 18 (CCL18) was first identified as a chemoattractant for naïve T cells. It has been reported to recruit T and B lymphocytes, and we show here, natural killer (NK) cells, but with low efficacy. Investigation of its ability to elicit G-protein-coupled signaling showed that it does not involve extracellular signal-regulated kinase (ERK) phosphorylation, and it is not able to induce receptor internalization, as assessed on CCR3. CCL18 has recently been reported to possess activities unrelated to cellular recruitment, but it had no effect on T lymphocyte proliferation. We postulated that a more potent chemoattractant may be produced under inflammatory conditions but only minor truncations were observed, with the major form being the full-length protein. In view of the lack of potent immunomodulatory properties, we wondered if binding to CCL18 by the tick chemokine binding proteins Evasin-1 and -4 was an artifact of the methods used, but complex formation was confirmed by size exclusion chromatography, and abrogation of its binding to, and antagonism of, CCR3. Its receptor has remained elusive since its cloning in 1997, although it has been reported to induce migration of breast cancer cells by signaling through PITPNM3, but we show that this receptor is not expressed on lymphocytes. We have developed a radiolabeled equilibrium competition binding assay and demonstrated that it bound with high affinity to peripheral blood leukocytes (PBLs), but the binding was displaced similarly by both unlabelled CCL18 as well as heparin. Both heparin binding and binding to PBLs are considerably abrogated by mutation of the BBXB motif in the 40s loop suggesting an essential role of the CCL18-glycosaminoglycan interaction.

## Introduction

Chemokines are cytokines with selective chemotactic properties. They coordinate leukocyte migration during immunity and inflammation and are also involved in the pathogenesis of several human diseases. The family consists of approximately 50 chemokines which trigger their biological responses by activating seven-transmembrane domain G-protein-coupled receptors (7TM GPCRs) on their target cells, 20 of which have been identified to date. By definition leukocyte chemoattraction is the hallmark function of chemokines, however chemokines also induce cellular responses that are unrelated to leukocyte migration such as cell differentiation and activation (1), apoptosis (2), development (3), angiogenesis (4), or tumor growth and metastasis (5, 6).

Two chemokines remain orphan in that their receptor has not yet been identified: CCL18 and CXCL14. CCL18 has been described to mediate various functions such as chemotaxis mediated by G-protein coupling, intracellular calcium mobilization, and actin polymerization, however its classical, signaling chemokine receptor(s), presumably a 7TM GPCR, remains elusive. It has, however, been reported to bind and antagonize CCR3 (7). Recently PITPNM3 was described to be a functional receptor for CCL18 (8). PITPNM3, which is also known as PYK2 N-terminal domain-interacting receptor 1 (Nir1), belongs to the phosphatidylinositol transfer protein (PITP) membrane-associated subfamily. PITPNM3/Nir1 was shown to be expressed in human retina, brain, spleen as well as ovary and recently it was also shown to be abundantly expressed in breast cancer cells (8, 9). CCL18 produced by breast tumor-associated macrophages was shown to be involved in breast cancer metastasis via the interaction and signaling through PITPNM3/Nir1, which was pertussis toxin (PTX) dependent, despite the fact that PITPNM3/Nir1 does not show apparent structural or functional similarity to conventional GPCRs (8).

We describe here chemotaxis induced by CCL18 on T lymphocytes as previously reported (10–12) as well as B lymphocytes (13) and show it also attracts natural killer (NK) cells. The response, which is particularly weak, is however PTX dependent, which points to the involvement of G_αi_ proteins and G-protein-coupled receptors.

Post-translational modifications of chemokines have been well described and have been shown to modulate chemokine activity with either no effect or decreased or increased activity, as well as altered receptor specificity (reviewed in 14). Modifications at the amino-terminus (N-terminus) of the chemokine have the greatest impact on biological activity and selectivity, due to the involvement of this region in the interaction with its receptor(s). Certain chemokines have been shown to be converted into potent chemoattractants by proteases, which are released during inflammatory responses (15). For example CCL5 is more potent in inducing CCR5 mediated responses when converted to 3–68, although this form becomes an antagonist on CCR1 (14). Importantly, low affinity ligands for CCR1, which are abundant in the circulation, are converted into high affinity ligands by processing at the N-terminus (14). In fact isolation of natural CCL18 from the conditioned medium of peripheral blood mononuclear cells (PBMCs) demonstrated variants lacking two or three N-terminal amino acid residues in addition to the intact full length CCL18 (16).

We have addressed several questions concerning the biological role of CCL18. We looked for a more potent form in the circulation under inflammatory conditions. Despite the fact that the chemoattractant properties of CCL18 are unimpressive, we addressed the question of whether the Evasins, chemokine binding proteins (CkBPs) produced by the tick *Rhipicephalus sanguineus*, really do bind CCL18. We also investigated whether the transmembrane receptor that mediates breast cancer cell metastasis is the elusive CCL18 receptor. Lastly, we considered attempting to identify the CCL18 receptor by expression cloning from responsive cells, but analysis of its binding to the surface of PBLs revealed that the major binding interaction is to glycosaminoglycans (GAGs).

## Materials and Methods

### Reagents

Chemokines were obtained from PeptroTech or produced as previously described (17). Polyclonal anti-CCL18 antibodies were obtained from R&D systems. Radiolabeled CCL18 (^125^I-CCL18) was obtained from Anawa. Evasin-1, -3, and -4 were produced as previously described (18, 19).

### Chemotaxis assays

Chemotaxis assays were carried out as previously described (20). Lymphocyte subpopulations (CD4^+^ T lymphocytes, CD8^+^ T lymphocytes, B lymphocytes, and NK cells) were purified from human blood by density gradient centrifugation through Ficoll followed by negative selection using a MACS Isolation kit (Miltenyi Biotec) following the manufacturer’s instructions. Twenty microliters of the cell solutions was deposited on top of the membrane of each well (75 × 10^3^ CD4^+^ and CD8^+^ T lymphocytes and NK cells and 50 × 10^3^ B lymphocytes). The chambers were incubated for 1 h.

### Chemotaxis assay with PTX-treated PBL

Peripheral blood leukocyte were purified by Ficoll gradient centrifugation (Ficoll-Paque PLUS, GE Healthcare) and separated from the monocytes by allowing them to adhere to tissue culture plastic in RPMI 1640 containing 10% Fetal Calf serum (FCS), 5 mM l-Glutamine, and 50 U/ml Penicillin/Streptomycin (P/S) for 120 min at 37°C. Fifty micrograms of PTX were suspended in 500 μl sterile ddH_2_O containing 2.5 mg/ml Bovine serum albumin (BSA) (100 μg/ml). Cells (30 × 10^5^) were suspended in RPMI 1640 complete medium containing 200 ng/ml PTX and incubated for 2 h in a 5% CO_2_ humidified incubator at 37°C. The PTX-treated cells were washed twice in chemotaxis medium and chemotaxis assays were performed as described above.

### ERK-1/2 phosphorylation

The experiment was performed as previously described (21) using T lymphocytes, which were purified from human blood by density gradient centrifugation through Ficoll followed by negative selection using a MACS Isolation kit (Miltenyi Biotec) following the manufacturer’s instructions.

### CCR3 ligand-induced internalization in eosinophils

Eosinophils were purified from human blood by negative immunomagnetic selection as previously reported (22) using the human Eosinophil Enrichment Kit (Easy Sep) following the manufacturer’s instructions. The cells were suspended in RPMI 1640 supplemented with 10% FCS, 5 mM l-Glutamine, and 50 U/ml P/S and stored overnight at 4°C. Eosinophils were exposed to 12.5 or 125 nM of CCL18 or 12.5 nM CCL5 and CCL11 for 15 min or 1 h as previously described (22). Cells were incubated with 1 μg anti-human CCR3 antibody (clone 5E8, BD Pharmingen) or isotype control (Alexa Fluor 647 Mouse IgG2b, gamma) and CCR3 expression analyzed by flow cytometry.

### Cell proliferation assay

Cellular proliferation was assessed using carboxyfluorescein diacetate succinimidyl ester (CFSE) and analyzed by flow cytometry as previously described (23). T lymphocytes were incubated with CFSE at a final concentration of 5 μM for 5 min at 37°C. The reaction was stopped by adding RPMI 1640 complete medium. CCL18 (1 nM, 10 nM, 100 nM, or 1 μM) was added to the cells 1 h before cell stimulation. After the pre-incubation period, cells were transferred to 96-well plates pre-coated with 5 μg/ml plate bound anti-CD3 (BD Pharmingen) in the presence of soluble anti-CD28 antibodies (10 μg/ml) (BD Pharmingen). In parallel the assay was performed without anti-CD3/CD28 stimulation.

### Generation of Δ2- and Δ3-CCL18-6His and ^44^AAGA^47^-CCL18

Mutagenesis was performed by Polymerase chain reaction (PCR) using a pEAK12d plasmid containing the sequence of CCL18-6His as template. The deletions of the N-terminal amino acids were made using site directed mutagenesis with the mutagenesis primer: Δ2-CCL18-6His_forward 5′-GCC CTC TGC TCC TGT GTT GGT ACC AAC AAA G and Δ2-CCL18-6His_reverse 5-CTT TGT TGG TAC CAA CAC AGG AGC AGA GGG C as well as Δ3-CCL18-6His_forward 5′-GGC CCT CTG CTC CTG TGG TAC CAA CAA AGA GC and Δ3-CCL18-6His_reverse 5′-GCT CTT TGT TGG TAC CAC AGG AGC AGA GGG CAA TG. The mutations of K44, R45, and R47 to alanine residues were performed using the primers: ^44^AAGA^47^_forward 5′-CCT CCT AAC CGC CGC CGG CGC CCA GAT CTG TGC TGA CCC C and ^44^AAGA^47^_reverse 5′-GGG GTC AGC ACA GAT CTG GGC GCC GGC GGC GGT TAG GAG G. The mutations were introduced in a one step PCR reaction followed by a *Dpn*1 digestion of the parental plasmid DNA.

### Protein purification and characterization

The mutant plasmids were transformed into HEK293-6E cells. Eight hundred milliliters of FreeStyle F17 Expression Medium supplemented with 4 mM l-Glutamine and 10 ml/l of 10% Pluronic F68 (final concentration 0.1%) were inoculated with HEK293-6E cells and grown in a shake flask on an orbital shaker at 80 rpm until a cell density between 1.5 and 2.0 × 10^6^ cells/ml was reached. Cells were transfected with 2 mg/l PEI and 1 mg/l plasmid. Cells were incubated at 37°C in a humidified incubator containing 5% CO_2_ for 5 days. The culture supernatant was harvested by centrifugation for 15 min at 3500 × *g* and filtered using 0.22 μm membrane filter device. The solution was adjusted to a pH between 7.0 and 7.2 and the solution was applied to an SP Sepharose column (XK50/4.6) previously equilibrated in 10 mM Tris/HCl, pH 7.0. Proteins were eluted with 1 M NaCl in 50 mM NaPO_4_, pH 7.3. The fractions containing the CCL18 proteins were pooled and applied to a POROS 20 MC metal chelate affinity column, which had previously been loaded with Ni^2+^ ions using a 100-mM Ni(II)SO_4_ solution and equilibrated with 50 mM NaH_2_PO_4_, 600 mM NaCl, 8.7% (vol/vol) glycerol, pH 7.5. Δ2-CCL18-6His, Δ3-CCL18-6His, and ^44^AAGA^47^-CCL18-6His were eluted by 50 mM NaH_2_PO_4_, 600 mM NaCl, 8.7% glycerol, 400 mM imidazole, pH 7.5. Protein containing fractions were loaded onto a Superdex 75 column (XK 16/60), which was previously equilibrated with PBS at a flow rate of 1.5 ml/min.

### Equilibrium competition binding assay

Binding assays were performed using PBL purified as described above. Cells were suspended at a density of 4 × 10^6^ cells/ml in binding buffer (50 mM Tris/HCl pH 7.5 buffer containing 1 mM CaCl_2_, 5 mM MgCl_2_, and 0.5% BSA) to be used at a final concentration of 0.1 × 10^6^ cells/well. ^125^I-chemokine was dissolved at 0.4 nM in binding buffer to reach 0.1 nM final concentration. Dilutions of CCL18 proteins were prepared by fourfold dilutions to cover a range from 10^−6^ to 10^−12^ M. The assay was performed in triplicate. The mixture of cells, chemokine, ^125^I-chemokine, and binding buffer was incubated for 4 h at room temperature (RT) under gentle agitation. Cells were then washed three times with 200 μl wash buffer (50 mM Tris/HCl pH 7.5 buffer containing 1 mM CaCl_2_, 5 mM MgCl_2_, 0.5% BSA, and 0.5 M NaCl) using vacuum filtration. Finally 50 μl of scintillation liquid was added to each well and the radioactivity measured using a β-scintillation counter. Binding assays were performed using the MultiScreen HTS 96-well filtration system.

### Surface enhanced laser desorption/ionization time of flight mass spectrometry

The surface enhanced laser desorption/ionization time of flight mass spectrometry (SELDI-TOF MS) platform was used in order to capture CCL18 on anti-CCL18 polyclonal antibody coated chips, which enables the analysis of biological samples unrelated to the complexity of the sample. Initially analysis of the integrity of CCL18 on the NP20 ProteinChip was first determined by applying CCL18 (5 μl at a concentration of 1 mg/ml) onto a NP20 ProteinChip Array (BioRad) as previously described (24). A saturated solution of sinapinic acid in 50% acetonitrile containing 1% Trifluoroacetic acid (TFA) in distilled H_2_O was added onto each spot and air-dried. Mass analyses were performed by SELDI-TOF MS using the ProteinChip Biology System II and the Ciphergen protein chip software version 3.2.1. Mass spectra were generated with a mass focus between 0 and 15 kDa. To determine the detection limit of CCL18 by SELDI-TOF MS a dilution series of recombinant CCL18 (0.15–10 ng final amount) in PBS, containing 0.1% Triton-X-100 was applied onto an anti-CCL18 polyclonal antibody coated RS100 ProteinChip Array (BioRad Laboratories) as previously described (24). A saturated solution of sinapinic acid in 50% acetonitrile containing 1% TFA in distilled H_2_O was added onto each spot, air-dried, and mass analysis were performed using the conditions as described above. For the detection of the chemokine in the synovial fluid RS100 ProteinChips coated with polyclonal anti-CCL18 antibodies were used. Due to the viscosity of the synovial fluid the samples were diluted in PBS, containing 0.1% Triton-X-100. The dilution of each sample was preformed according to the concentration of CCL18 in the synovial fluid determined by Luminex analysis using the Custom Human 27-Plex Cytokine Panel according to the manufacturer’s instructions. The synovial fluid samples were diluted to load a final amount of 1 ng CCL18 on the RS100 ProteinChip.

### Size exclusion chromatography

Complex formation of CCL18 with Evasin-1, -3, and -4 was analyzed by size exclusion chromatography (SEC). Five hundred micrograms of CCL18 and an equimolar amount of the Evasins and were incubated for 45 min at RT in PBS and then applied to a Superdex75 column, previously equilibrated with PBS and calibrated with the following proteins: conalbumin, 75 kDa; ovalbumin, 44 kDa; carbonic anhydrase, 29 kDa; ribonuclease A, 13.7 kDa, and aprotinin, 6.5 kDa. Fractions were eluted at a flow rate of 1.5 ml/min and were subsequently analyzed on silver stained sodium dodecyl sulfate polyacrylamide gel electrophoresis (SDS-PAGE) gels.

### Neutralization of bioactivity

Inhibition of ^125^I-CCL18 binding to PBLs by the Evasins was assessed as described above. Neutralization of inhibition by CCL18 of CCL11 induced chemotaxis was performed using the EC_80_ (the concentration of CCL11, producing 80% of the maximal migration) and the CCL18 at a concentration at which a maximal inhibitory effect was observed. The neutralizing activity of the Evasins was tested by incubating serial dilutions to cover a range from 10^−11^ to 20^−6^ M. CCL11, CCL18, and the Evasins were placed in the lower chamber of the chemotaxis plate in triplicate. The conditions for the assay were used as described above.

### Determination of mRNA levels by quantitative PCR

Total RNA was isolated from the cells of interest using a RNeasy microkit (Qiagen) and reverse-transcribed using a iScript cDNA Synthesis Kit (BioRad). The cDNA quality control was performed by the amplification of the housekeeping gene (HKG) glyceraldehyde 3-phosphate dehydrogenase (GAPDH). The PCR reaction was performed using the Platinum Blue PCR SuperMix and the GAPDH_forward 5′-CTG CAC CAC CAA CTG CTT AG and reverse primer GAPDH_reverse 5′-CCA GTG AGC TTC CCG TTC AG according to the manufacturer’s instructions. The amplified PCR products were analyzed by agarose gel electrophoresis using a 1.1% agarose gel. Quantitative real time PCR was performed using a QuantiFast SYBR green PCR kit (Roche) according to the manufacturer’s instructions and PCR products were detected using an ABI PRISM 7900HT sequence detection system instrument (Applied Biosystems). Validated primer pairs were used to amplify PITPNM3/Nir1 (QT00493675) from Qiagen. GAPDH (QT01192646), actin (QT01680476), and 18S ribosomal RNA (18S rRNA) (QT00199367) expression was used as HKGs. The SDS 2.2.2 software system (Applied Biosystems) was used to analyze the expression levels of the target genes. The relative expression of the target gene PITPNM3 was quantified in relation to the HKGs GAPDH, actin, and 18S rRNA. The relative quantification ratios (*R*) were calculated in % of HKG using the following equation, in which an optimal doubling of the target DNA during each performed PCR cycle is assumed: *R* = 100/2^(CtPITPNM3 − Ct HKG)^.

### Chemokine binding assay to immobilized heparin

To test the ability of CCL18 and ^44^AAGA^47^-CCL18 for their heparin binding ability a colorimetric enzyme-linked immunosorbent assay was performed as previously described using polyclonal anti-CCL18 antibodies (20). For detection a secondary antibody solution (donkey anti-goat IgG-HRP antibody) at 1:10,000 was used.

## Results

### Chemotactic responses induced by CCL18

**Figure 1:**
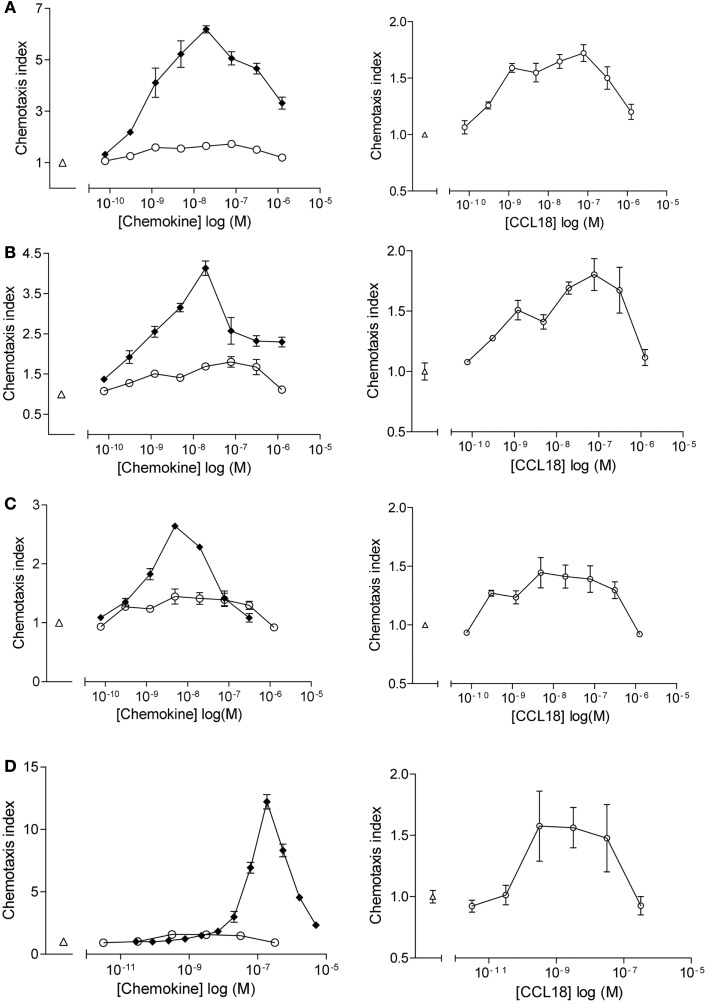
***In vitro* chemotactic responses of lymphocyte subpopulations to CCL18**. Chemotactic responses of **(A)** CD4^+^ T lymphocytes, **(B)** CD8^+^ T lymphocytes, **(C)** NK cells, and **(D)** B lymphocytes induced by CCL18 (○) or mediated by potent receptor agonists. **(A,B)** CXCL12α, **(C)** extracellular domain of CX_3_CL1, and **(D)** CXCL13. Medium was used as a control (Δ). Increase in scale and exclusion of chemotactic response induced by the potent receptor agonists (right panel). Data are expressed as chemotaxis index ± SEM. Data points are in triplicate. One out of two independent experiments is shown.

Chemotactic responses of purified leukocyte subpopulations (CD4^+^ T lymphocytes, CD8^+^ T lymphocytes, B lymphocytes, and NK cells) towards CCL18 displayed bell-shape curves with a maximal response induced by 10–100 nM CCL18 (Figure 1). The chemotactic index was generally approximately 0.5, although a chemotactic index of 3 was sometimes achieved whilst cells from other donors showed almost no response. The chemotactic responses induced by CCL18 are weak compared to the lymphocyte chemoattractants CXCL12α, CXCL13, and CX_3_CL1, which were used as positive controls for T lymphocytes, B lymphocytes, and NK cells, respectively. CXCL12α induced a chemotactic response of T lymphocytes with a chemotactic index between 3 and 6 (Figures 1A,B, lefts panels). A chemotactic index around 3 was obtained for NK cells migrating to CX_3_CL1 (Figure 1C, left panel) and B lymphocytes showed a chemotactic response to CXCL13 with a chemotactic index of 12.5 (Figure 1D, left panel).

The chemotactic response of freshly isolated monocytes, as well as monocytes cultured between 1 and 4 days as described (25), towards CCL18 was tested. No chemotactic response of freshly isolated or cultured monocytes to CCL18 was obtained (data not shown). Similarly no chemotactic response of the monocytic cell line THP1, nor of the T cell lines, MOLT4, and Jurkat to CCL18 was observed (data not shown).

Based on our results PBL, comprising T lymphocytes, B lymphocytes, and NK cells, compose the leukocyte subpopulations responding to CCL18 and were used in most of the following experiments.

### Investigation of signal transduction pathways induced by CCL18

**Figure 2:**
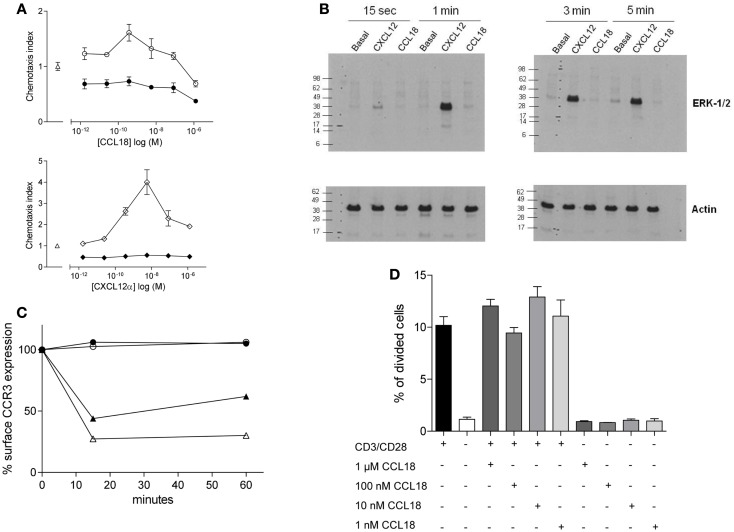
**Ability of CCL18 to induce signal transduction pathways and influence cell proliferation**. One out of three independent experiments from separate donors is shown. **(A)** *In vitro* chemotactic responses of PBL mediated by CCL18 imply G_iα_-protein-coupled signal transduction mechanism. Chemotactic responses of PBL induced by CCL18 (○). Influence of PTX (200 ng/ml) on CCL18-mediated (●) chemotactic responses (upper panel). Chemotactic response mediated by CXCL12α (◊) and influence of PTX on CXCL12α-mediated responses (♦) (lower panel). Medium was used as a control (Δ). Data are expressed as chemotaxis index ± SEM. Data points are in triplicate. **(B)** ERK-1/2 phosphorylation analysis in T lymphocytes after stimulation with CCL18. Western Blot analysis using an anti-phosphor p44/42 MAPK (ERK-1/2) antibody. Time course for ERK-1/2 phosphorylation in T lymphocytes stimulated with 100 nM CCL18 or CXCL12α. Detection of actin was used as a loading control. **(C)** CCR3 cell surface expression after CCL18 stimulation of human eosinophils. Human eosinophils were stimulated with 12.5 nM CCL18 (○), CCL5 (▲), CCL11 (Δ), or 125 nM CCL18 (●) for 15 or 60 min. Ligand-induced CCR3 internalization was measured by Flow cytometry. Surface expression of CCR3 was compared with CCR3 expression of unstimulated eosinophils. Results are expressed in % of CCR3 surface expression. **(D)** T lymphocyte proliferation analysis after CCL18 stimulation. T lymphocytes were stimulated or not with anti-CD3/CD28 antibodies in the presence or absence of an increasing concentration of CCL18 (1 nM, 10 nM, 100 nM, or 1 μM). The division of cells was measured by CFSE and flow cytometry. Data points are in triplicate. Data are expressed in % of divided cells ± SEM.

We tested whether the chemotactic responses induced by CCL18 are G_iα_ protein mediated as previously reported (10, 13) by pre-treatment of the cells with PTX, which is a broad inhibitor of G_i_ protein-coupled receptors. This resulted in the complete abrogation in chemotactic responses of the cells to CCL18 (Figure 2A, upper panel), indicating a G-protein-coupled receptor-mediated event. The chemotaxis induced by CXCL12α and the influence of PTX were used as a positive control (Figure 2A, lower panel). Cell viability was determined by flow cytometry upon PTX-treatment of the cells using Propidium iodide. No discrepancy in cell viability was observed between PTX-treated and untreated cells (data not shown).

Next we investigated the ability of CCL18 to induce extracellular signal-regulated kinase (ERK)-1/2 phosphorylation in T lymphocytes, as the activation of the ERK-1/2 signaling pathway is known to be involved in some chemokine induced responses (26, 27). T lymphocytes were stimulated with 100 nM CCL18, and CXCL12α was again used as a positive control. A time course was performed ranging from 15 s to 5 min of stimulation. CXCL12α induced ERK-1/2 phosphorylation at all time points tested. However the stimulation with CCL18 did not result in ERK-1/2 phosphorylation at any time point (Figure 2B). The ERK-1/2 phosphorylation in CD4^+^ and CD8^+^ T lymphocytes was tested using different concentrations of CCL18 (10, 50, 100, and 1 μM) and a broader time course ranging from 15 s to 15 min. However at all concentrations of CCL18 and at all time points no induction of ERK-1/2 phosphorylation was detected (data not shown).

We then investigated whether CCL18 induces other signaling pathways such as that which is required for receptor internalization. CCL18 has been reported to bind, and antagonize CCR3 (7), so we addressed the question of whether it was able to induce the signaling pathways for receptor internalization. Receptor internalization studies were performed using freshly isolated eosinophils, on which CCR3 has been described to be the major chemokine receptor. The down-modulation of the CCR3 surface expression as determined by flow cytometry analysis upon the exposure of eosinophils with CCL5 and CCL11 was used as a positive control (22). The exposure of eosinophils to CCL5 caused a reduction of CCR3 receptor surface expression of 56.1 and 38.03%, and CCL11 caused CCR3 internalization of 72.7 and 69.8% after 15 and 60 min, respectively. However there was no reduction in CCR3 surface expression observed after the exposure of eosinophils to CCL18 (12.5 or 125 nM) after 15 or 60 min, indicating that CCL18 does not trigger the signaling pathways to induce this activity (Figure 2C).

We then investigated whether CCL18 exhibits mitogenic activity inducing cell proliferation. T lymphocytes were stimulated by mimicking the binding of antigen presenting cells using anti-CD3 and anti-CD28 antibodies for 72 h. In order to investigate the mitogenic activity of CCL18 on T lymphocyte proliferation the cells were stimulated with increasing concentrations of CCL18 in the presence or absence of anti-CD3 and anti-CD28 antibodies. The quantification of cell proliferation was estimated based on the number of cell division using CFSE and flow cytometry assessment. Figure 2D shows an increase in T lymphocyte proliferation in response to CD3/CD28 costimulation, but no effect on T lymphocyte proliferation was observed upon stimulation with CCL18.

### Characterization of Δ2- and Δ3-CCL18

**Figure 3:**
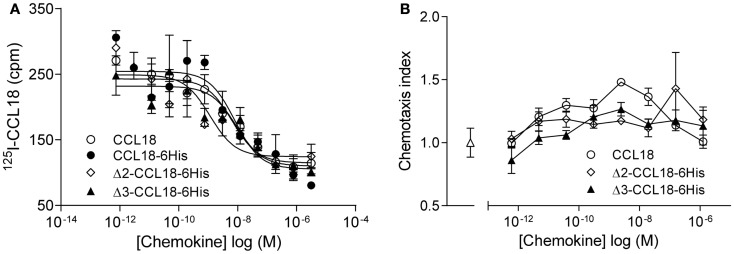
**Characterization of Δ2-CCL18 and Δ3-CCL18**. **(A)** Equilibrium competition binding assay using ^125^I-CCL18 on PBL. Binding of ^125^I-CCL18 was competed using an increasing concentration of CCL18 (IC_50_: 6.44 nM), CCL18-6His (IC_50_: 7.89 nM), Δ2-CCL18-6His (IC_50_: 1.14 nM), Δ3-CCL18-6His (IC_50_: 9.04 nM) with equal potency. Data are expressed in cpm ± SEM. Data points are in triplicate. The graph represents a single experiment. **(B)** *In vitro* chemotactic response of PBL to CCL18 and the N-terminal truncated forms Δ2-CCL18-6His and Δ3-CCL18-6His. Medium (Δ) was used as a control. Data are expressed as chemotaxis index ± SEM. Data points are in triplicate. One representative experiment out of three is shown.

Since N-terminal processed forms of CCL18 have previously been identified from the conditioned medium of PBMC (16), but had not been biologically characterized, we produced these truncated forms in order to determine potential differences in biological activity. Equilibrium competition binding assays were performed on PBL using ^125^I-CCL18 as tracer. CCL18 and CCL18-6His competed for ^125^I-CCL18 binding to PBL with IC_50_ values of 6.44 and 7.89 nM, respectively. The IC_50_ for Δ2-CCL18-6His was 1.14 nM and for Δ3-CCL18-6His 9.04 nM. Thus similar potencies were obtained for the CCL18 truncated forms demonstrating no significant difference in receptor binding affinity (Figure 3A). The lack of impact on activity by these two truncations was confirmed by chemotaxis assays, where the chemotactic responses for the Δ2- and Δ3-CCL18-6His forms were similar to the full-length CCL18 protein (Figure 3B).

### SELDI-TOF MS analysis of CCL18 expression

**Figure 4:**
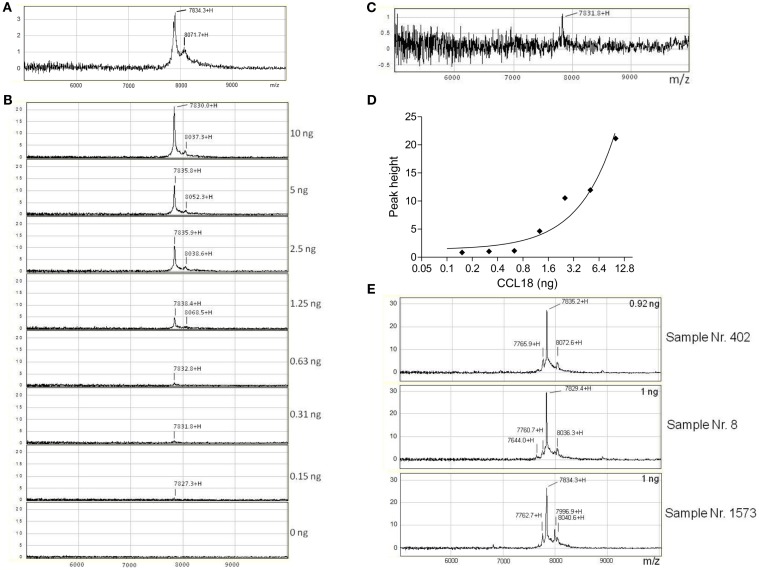
**Detection and identification of processed CCL18 isoforms in the synovial fluid of RA and OA patients by SELDI-TOF MS**. **(A)** MS spectra of recombinant CCL18 determined using NP20 ProteinChips. Recombinant CCL18 (100 ng) was applied onto a NP20 ProteinChip Array and mass analyses were performed by SELDI-TOF MS. **(B)** Standard curve of recombinant CCL18 with RS100 ProteinChips. Dilutions of recombinant CCL18 ranging from 0.15 to 10 ng were applied onto RS100 ProteinChip Arrays containing immobilized polyclonal anti-CCL18 antibodies. **(C)** Increase in scale at 0.31 ng. **(D)** Peak height displayed according to amount of recombinant CCL18. **(E)** RS100 ProteinChips were coated with polyclonal anti-human CCL18 antibodies and incubated with synovial fluid from RA (Sample number 402 and 8) or OA (Sample number 1573) patients. Mass spectra were generated by SELDI-TOF MS. Spectra are representative for four RA and OA patient samples analyzed.

In order to identify whether other truncated forms of CCL18 exist during inflammation we isolated CCL18 from the synovial fluid of rheumatoid arthritis (RA) patients using SELDI-TOF MS. Initially the integrity of recombinant CCL18 protein samples was verified on the NP20 ProteinChip. CCL18 was intact with a mass-to-charge ratio (m/z) of 7834.3 (MW_theo_ 7851.19 Da) (Figure 4A). In parallel to the SELDI-TOF MS analyses the mass of recombinant CCL18 was also analyzed by Matrix-assisted laser desorption/ionization time of flight mass spectrometry (MALDI-TOF MS). A mass of 7850.80 Da was obtained, almost identical to the theoretical molecular weight of CCL18 (data not shown). This difference in mass observed between the two mass spectroscopic technologies was consistently observed and is due to the lower resolution and mass accuracy with SELDI-TOF MS. This lower accuracy in the determination of the molecular mass might be due to a short time of flight resulting in a less precise molecular weight determination. In addition to the peak corresponding to full length CCL18 a second, non-identified peak with a m/z of 8071.7 was observed, thus 237.4 Da higher, and which was present in all subsequent analyses.

To detect truncated forms of CCL18 in biological fluids affinity capture by polyclonal anti-CCL18 antibodies is required. Polyclonal anti-CCL18 antibodies were immobilized on a RS100 Protein Chip by the covalent binding to pre-activated carbonyl diimidazole functional groups on the chip. After the unreacted groups were blocked with ethanolamine, the ProteinChip spots were incubated with dilutions of recombinant CCL18 ranging from 0.15 to 10 ng in order to determine the detection limit of CCL18 (Figure 4B). CCL18 was detected with an average m/z of 7833 ± 1.45, corresponding to the mass obtained by the analysis of recombinant CCL18 on NP20 ProteinChips. The signal intensity correlates to the amount of recombinant CCL18 and demonstrated a limit of detection at 0.31 ng (3.1 ng/ml) (Figure 4C). The peak height correlating to the amount of recombinant CCL18 is shown in Figure 4D. In all analyses a mass 8049 ± 7.28 was obtained, corresponding to the non-identified mass previously observed (Figure 4A).

In order to detect CCL18 using the SELDI-TOF MS analysis a sufficient amount of CCL18 present in the synovial fluid needed to be ensured. Therefore CCL18 levels were determined in the synovial fluid samples by Luminex analyses (data not shown). CCL18 was one of the most abundant chemokines in the synovial fluid among different chemokines measured (A. Garin et al., manuscript in preparation). The level of CCL18 in the synovial fluid samples of RA patients was 574.9 ± 109 ng/ml (*n* = 21). In the synovial fluid samples of Osteoarthritis (OA) patients CCL18 levels of 307 ± 92.5 ng/ml (*n* = 6) were detected. Thus the amounts of CCL18 in the samples of RA patients was about twofold higher than the levels found in samples of OA patients. These analyses revealed that the amount of CCL18 in the synovial fluid is sufficient for the detection by SELDI-TOF MS analysis.

Since synovial fluid is particularly viscous the samples were diluted to a CCL18 concentration of 10 ng/ml, allowing each spot to be incubated with synovial fluid containing a final amount of 1 ng CCL18. Some samples were measured in duplicate if the amount of sample was sufficient. The differences in the m/z values of each protein signal were compared to full length CCL18 within one sample (Δ to full length CCL18). The samples of four RA and OA patients were analyzed and the mass values of the protein-signals obtained from the SELDI-spectra are summarized in Table 1.

**Table 1:** **Summary of the mass detected in the synovial fluid samples of RA and OA patients by SELDI-TOF MS**.

Sample number	ng/ml	MW (Da)		Δ To full length CCL18	
**RHEUMATOID ARTHRITIS PATIENTS**					
8	41.1	7644.0 + H	7614.5 + H	−185.4	−185.3
		7760.7 + H	7729.6 + H	−68.7	−70.2
		**7829.4 + H**	**7799.8 + H**
		8036.9 + H	8011.3 + H	+207.5	+211.5
385	389.5	7764.6 + H	7766.5 + H	−69.8	−73.0
		**7834.4 + H**	**7839.5 + H**		
		8041.2 + H	8046.0 + H	+206.8	+206.5
402	92.01	*7655*.*3* + *H*		−*179*.9	
		7765.9 + H	7766.3 + H	−69.3	−72.2
		**7835.2 + H**	**7838.5 + H**		
		8040.8 + H	8043.9 + H	+205.6	+205.4
2282	609.7	7747.9 + H	7739.8 + H	−72.4	−71.8
		**7820.3 + H**	**7811.6 + H**		
		8029.1 + H	8026.9 + H	+208.8	+215.3
**OSTEOARTHRITIS PATIENTS**					
669	54.0	7766.6 + H	7765.5 + H	−71.3	−71.2
		**7837.9 + H**	**7836.7 + H**		
		8001.6 + H	7999.5 + H	+163.7	+162.8
		8046.6 + H	8042.8 + H	+208.7	+206.1
1201	58.7	7761.0 + H	7761.9 + H	−70.6	−70.1
		**7831.6 + H**	**7832.0 + H**		
		7993.1 + H	7994.9 + H	+161.5	+162.9
		8034.7 + H	8036.7 + H	+203.1	+204.7
1573	37.0	7762.7 + H		−71.6	
		**7834.3 + H**			
		7996.9 + H		+162.6	
		8040.6 + H		+206.3	
2076	591.0	7719.5 + H		−72.4	
		**7791.9 + H**			
		8002.9 + H		+211.0	

*The concentration of CCL18 in each sample is indicated in ng/ml, as well as the mass obtained from each sample [MW (Da)] and the discrepancy of mass to full length CCL18 which is highlighted in bold (Δ to full length CCL18)*.

The spectra revealed a major peak with an average of 7827 ± 4.05 Da (Figure 4E) corresponding to the mass obtained for recombinant CCL18. A second peak approximately 200 Da higher than that of CCL18, with an average of 8034 ± 3.47 Da was detected in all synovial fluids tested. This peak correlates to the non-identified peak, which was also observed using recombinant CCL18 (Figure 4A). An additional peak with an average in molecular weight of 7765 ± 4.1 with a 10-fold lower signal intensity was detected in all synovial fluids tested. This peak revealed a molecular weight of 71 Da less than full length CCL18, corresponding to the theoretical mass of CCL18 lacking an alanine residue. As CCL18 has an alanine residue at both N- and C-termini the truncation could be at either position. In the synovial fluid samples of two RA patients (sample number: 8 and 402) a second truncated form, with an average mass off 7638 ± 12.16 thus lacking approximately 189 Da was detected (Figure 4E), corresponding to the mass of an alanine-asparagine dipeptide of 185.19 Da. Thus this corresponds to the truncated form of CCL18 lacking the C-terminal dipeptide, suggesting that the 70-Da truncation occurs at the C-terminus. Interestingly an additional peak with an average of 7997 ± 1.53 Da was observed in three out of four synovial fluid samples tested from OA patients (Figure 4E, sample number 1573). It is highly unlikely that CCL18 is posttranslationally glycosylated, as there are no predicted N- and O-glycosylation sites. However as the principal intention of the SELDI-TOF MS analysis was the identification of putative N-terminal truncated forms of CCL18 with increased potency, no further investigations regarding the observed modified forms were undertaken, and the nature of this modification remains to be elucidated. Lastly, besides differences in the masses, the intensity of the protein-signal allows a comparison of the protein levels. The signal intensity of full length CCL18 was predominant in all the synovial fluid samples tested, indicating that this form is the biologically relevant species.

### The tick CkBPs Evasin-1 and -4 bind CCL18

An interaction of CCL18 with Evasin-1 (19) and -4 (28) has been shown using the surface plasmon resonance method. In order to confirm this interaction Dot-Blot and SEC analyses were performed. Dot-Blot analyses were positive for Evasin-1 and -4, but negative for Evasin-3 as expected, thereby providing a second line of evidence that these CkBPs do bind CCL18 (data not shown).

**Figure 5:**
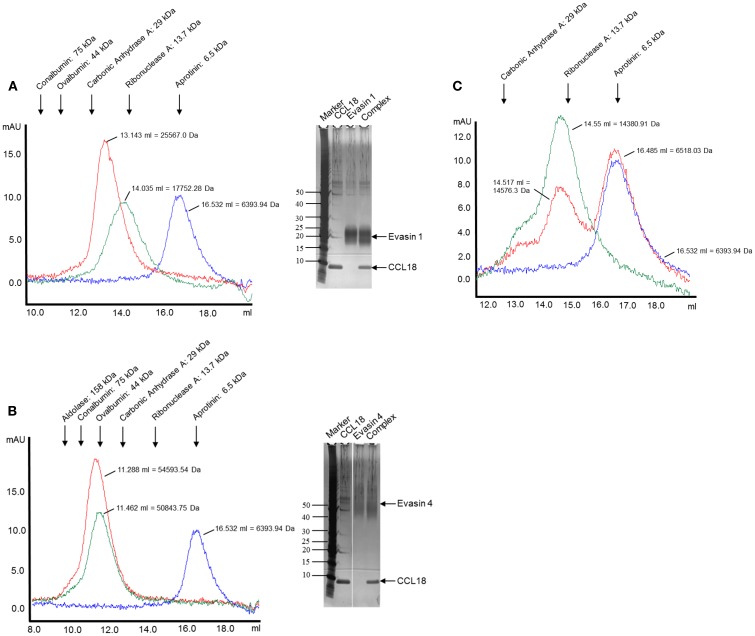
**Analysis of complex formation of Evasin-1, -3, and -4 with CCL18 by SEC**. CCL18 was incubated with an equimolar amount of **(A)** Evasin-1, **(B)** -4, and **(C)** -3 and injected onto an analytical Superdex 75 Prepgrade 10/300GL Tricorn column. The elution profile of CCL18 (blue), Evasins (green), and the mixture of CCL18 and Evasins (red) is shown in milliabsorbance units (mAU). The elution volume and corresponding molecular weight are indicated for each peak. The elution volume of each protein used for calibration is indicated on the top according to their molecular weights. **(A,B)** SDS-PAGE analyses were performed with the fractions corresponding to the peak elution fractions. SDS-PAGE stained with silver. One representative experiment out of two is shown.

The complex formation was further assessed by analyzing the elution profiles of CCL18 incubated with an equimolar amount of the Evasins by SEC. As shown in Figure 5, CCL18 eluted at a volume corresponding to a monomeric topology. The discrepancy in the theoretical masses and those determined by SEC for Evasin-1 (Figure 5A) and -4 (Figure 5B) is due to the glycosylation of the proteins and possibly to non-spherical structures. After the injection of the Evasin-1/CCL18 (Figure 5A) and Evasin-4/CCL18 (Figure 5B) complexes the proteins co-eluted in a single peak with an elution volume corresponding to molecular masses of 25,600 and 54,600 Da, respectively, indicating a 1:1 stoichiometry of the complex.

The presence of the two proteins in the peak was confirmed by SDS-PAGE analysis (Figures 5A,B, right panels). On the contrary, and again as expected, the mixture of Evasin-3 and CCL18 eluted as two discrete peaks at the same volume as for the individual proteins (Figure 5C). This biochemical analysis again confirmed the selective binding of CCL18 by Evasin-1 and -4.

**Figure 6:**
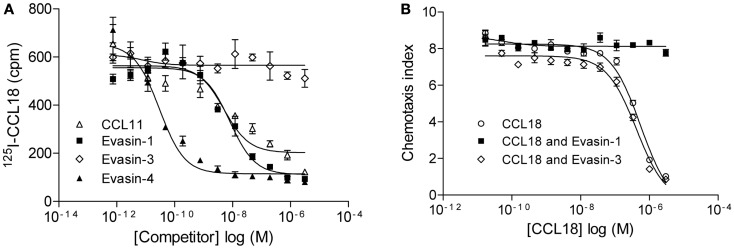
**Evasin-1 neutralizes the antagonistic effect of CCL18 on CCR3**. **(A)** Equilibrium competition binding assay of ^125^I-CCL18 on L1.2/CCR3 transfectants using an increasing concentration of CCL11 and the CCL18-binding proteins Evasin-1, -3, and -4. Data are expressed in cpm ± SEM. Data points are in triplicate. Inhibitory activity of CCL11 (IC_50_: 4.99 nM), Evasin-1 (IC_50_: 7.94 nM), and -4 (IC_50_: 0.03 nM) on ^125^I-CCL18 binding to CCR3. **(B)** Inhibition of chemotaxis using L1.2/CCR3 transfectants. Migration of L1.2/CCR3 transfectants to a constant concentration of 1 nM CCL11 was inhibited by an increasing concentration of CCL18 (IC_50_: 556.8 nM). The effect of Evasin-1 on the antagonistic activity of CCL18 on CCR3 was assessed by the incubation of CCL18 with a twofold access of Evasin-1. Evasin-3 (IC_50_: 421 nM) was used as a control. Data are expressed as chemotaxis index ± SEM. Data points are in triplicate. One representative experiment out of two is shown.

The relevance of this interaction was then assessed by the ability of Evasin-1 and -4 to compete for ^125^I-CCL18 binding to CCR3 (Figure 6A). The binding of ^125^I-CCL18 to CCR3 was inhibited by Evasin-1 and -4 with IC_50_ values of 9.92 ± 1.98 nM (*n* = 2) and 0.015 ± 0.02 nM (*n* = 2), respectively. Again, Evasin-3 did not inhibit ^125^I-CCL18 binding to CCR3 (Figure 6A). The neutralization ability of this interaction was corroborated by the abolition of the inhibition of CCR3-mediated responses by CCL18 (7, 29). CCL18 inhibits the chemotactic response of L1.2/CCR3 transfectants induced by 1 nM CCL11 with an IC_50_ of 317.6 ± 48.77 nM (*n* = 7), and this inhibition is abrogated in the presence of Evasin-1 (Figure 6B). The inhibitory effect of 1 μM CCL18 which completely inhibits migration to CCL11 was neutralized in the presence of ≥1 μM Evasin-1 (data not shown). A constant dose of 1 nM CCL11 was then incubated with an increasing concentration of CCL18 or CCL18 previously incubated with a twofold molar excess of Evasin-1 or -3 (Figure 6B). Evasin-1 completely abrogated the antagonistic effect of CCL18. Evasin-3 did not show any effect on the antagonistic activity of CCL18 [IC_50_: 446.5 ± 105 nM (*n* = 3)]. Evasin-4 could not be analyzed in this experiment since it inhibits the response directly by binding CCL11.

### Analysis of PITPNM3/Nir1 expression in leukocytes

PITPNM3/Nir1 was recently described to be a signaling receptor for CCL18 on breast cancer cells (8). Therefore we aimed to investigate whether PITPNM3/Nir1 is responsible for CCL18-dependent responses on leukocytes. The mRNA expression level of PITPNM3/Nir1 was determined by quantitative PCR (qPCR) analysis from different cell subpopulations: B lymphocytes, naïve B lymphocytes, T lymphocytes, CD8^+^ T lymphocytes, naïve CD4^+^ T lymphocytes, Monocytes, and NK cells. Universal Human Reference RNA was used as a positive control for the detection of PITPNM3/Nir1 mRNA expression. The mRNA expression levels were quantified against the HKGs GAPDH, actin, and 18S rRNA. PITPNM3/Nir1 mRNA was detected in the Universal Human Reference RNA control, whereas no expression of PITPNM3/Nir1 mRNA was observed in the various cell subpopulations tested (data not shown).

**Figure 7:**
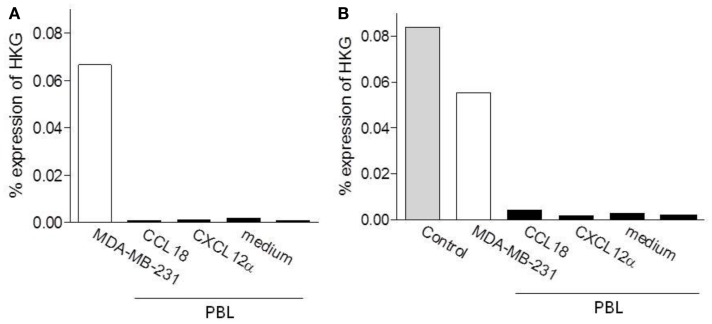
**Determination of the PITPNM3/Nir1 mRNA expression level in CCL18 responsive cells**. Analysis of PITPNM3/Nir1 mRNA expression by qPCR with **(A)** 1.25 ng and **(B)** 3.13 ng cDNA per well. Universal Human Reference RNA (Control) and MDA-MB-231 were used as positive controls. CCL18: PBL upon migration to 10 nM CCL18; CXCL12α: PBL upon migration to 10 nM CXCL12α and medium: PBL collected in the lower chemotaxis well without the addition of chemokine. Data are expressed in % expression of HKG (GAPDH, actin, 18S rRNA) ± SEM. The graphs represent a single experiment.

In order to determine if PITPNM3/Nir1 mediates CCL18 induced cell migration an enrichment of PITPNM3/Nir1 expressing cells would be obtained upon migration to CCL18. Thus we then assessed the PITPNM3/Nir1 mRNA expression level in PBL upon migration to CCL18 in two separate experiments. Chemotaxis assays were performed using PBL migrating to 10 nM CCL18, following subsequent isolation of total RNA and qPCR analysis were performed (Figure 7). As described above the mRNA expression levels were quantified against the HKGs GAPDH, actin, and 18S rRNA. The PITPNM3/Nir1 expression level of the cells upon migration to CCL18 was compared to the expression level in the cell population of origin as well as in cells upon migration to 10 nM of the control chemokine CXCL12α and the cells collected in the lower chemotaxis wells without the addition of chemokine. Total RNA isolated from the PITPNM3/Nir1 expressing breast cancer cell line MDA-MB-231, served as a control for the detection of PITPNM3/Nir1 expression. In addition Universal Human Reference RNA was used as a positive control for PITPNM3/Nir1 mRNA detection in the second experiment. PITPNM3/Nir1 expression was not detected in PBL and no enrichment in the PITPNM3/Nir1 mRNA expression level was obtained in cells upon CCL18 migration compared to the cells migrating in response to medium alone. A small increase in mRNA expression in cells migrating to CCL18 was apparent in the second experiment but no PITPNM3/Nir1 protein was observed by Western blot analysis using PBL protein extracts, whilst strong expression in the MDA-MB-231 cells was observed (data not shown). This leads to the conclusion that PITPNM3/Nir1 does not trigger CCL18-mediated chemotactic responses of leukocytes.

### Equilibrium competition binding assay to PBLs

**Figure 8:**
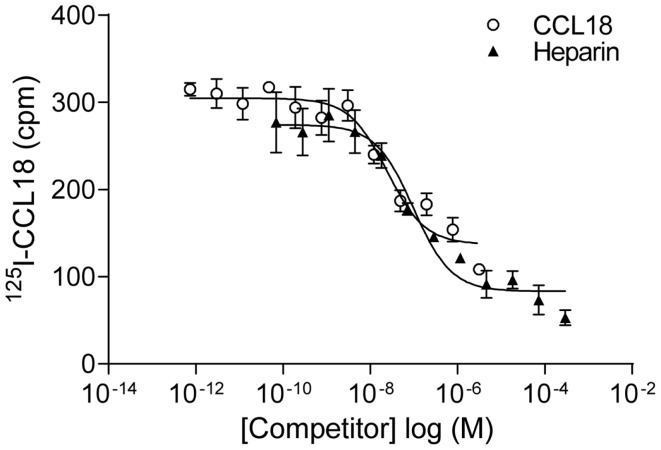
**Equilibrium competition binding assays of ^125^I-CCL18 on PBL**. The binding of ^125^I-CCL18 to PBL was competed with Heparin (heparin-3393) (IC_50_: 97.43 nM) and CCL18 (IC_50_: 24.37 nM). Data are expressed in cpm ± SEM. Data points are in triplicate. One representative experiment out of six is shown.

In order to identify the receptors binding to CCL18 we aimed to use an expression cloning strategy, which required an assay in order to detect the presence or absence of the receptor(s) binding to CCL18. Equilibrium competition binding assays were developed on PBL using ^125^I-CCL18. Unlabelled CCL18 displaced the tracer with an IC_50_ of 61.87 ± 38.09 nM (*n* = 9) (Figure 8). However as chemokines also interact with the GAGs on the cell surface the binding of ^125^I-CCL18 on PBL was competed with heparin. An IC_50_ of 246.4 ± 89.49 nM (*n* = 6) was obtained. Importantly, both CCL18 and heparin caused almost total displacement of the bound ^125^I-CCL18. This demonstrated a binding capacity with only fourfold difference in affinity between the CCL18-receptor and CCL18-GAG interaction, which would not enable subsequent screening in an expression cloning approach.

### Identification and characterization of the CCL18-GAG-binding epitope

**Figure 9:**
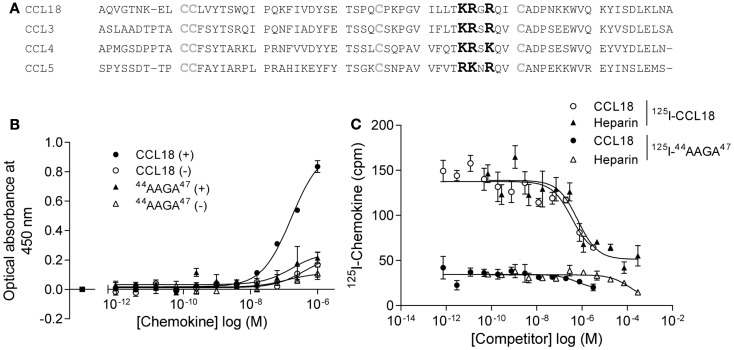
**Identification of the BBXB GAG-binding motif in the 40s loop of CCL18**. **(A)** Prediction of the BBXB GAG-binding motif of CCL18 by a sequence alignment of CCL18 with CCL3, CCL4, and CCL5. Basic residues, which are part of the BBXB motif, are highlighted in bold and the conserved cysteine residues in gray. **(B)** Comparison of the ability of CCL18 and ^44^AAGA^47^-CCL18 to bind to heparin immobilized on Epranex plates. Immobilization of a dilution series of CCL18 and ^44^AAGA^47^-CCL18 (^44^AAGA^47^) onto a heparin coated (+) or uncoated (−) heparin binding plate. Wells containing no chemokine were used as a control (■). Data are expressed in optical absorbance at 450 nm. Data points are in duplicate. One representative experiment out of three is shown. The capacity of ^44^AAGA^47^-CCL18 to bind to heparin is reduced. **(C)** The binding of ^125^I-CCL18 and ^125^I-^44^AAGA^47^-CCL18 (^125^I-^44^AAGA^47^) to PBL was competed with Heparin (heparin-3393) and CCL18. Data points are in triplicate. The mutation of the basic amino acids in the BBXB motif of CCL18 into alanine residues considerably abrogates binding to PBL.

In order to limit binding of CCL18 to GAGs we constructed a mutant designed to abrogate interaction with GAGs. Previous studies on heparin binding proteins have shown that the binding to GAGs is mediated by clusters of basic residues in the protein (30). CCL18 is a basic chemokine with an isoelectric point of pH 9.21. The inspection of the primary structure of CCL18 exhibits two separate clusters of basic residues, ^44^KRGR^47^ and ^55^KKWVQK^60^. The first cluster is located in the 40s loop and the second at the C-terminal region merging from the 50s loop into the C-terminal α-helix. Since the BBXB motif is a well characterized heparin binding motif, which has been previously shown to play this role on the 40s loop of related chemokines, CCL3 (31, 32), CCL4 (33), and CCL5 (34, 35) we created the variant ^44^AAGA^47^-CCL18 (Figure 9A).

The binding of ^44^AAGA^47^-CCL18 to heparin was investigated using heparin coated binding plates (Figure 9B). The amount of protein bound to immobilized heparin was determined using polyclonal anti-CCL18 antibodies. Non-specific interactions of the proteins with the plate were determined by directly measuring the interaction of the protein with the uncoated plate. The mutant showed 85% reduction in binding capacity in comparison to CCL18, confirming the involvement of the ^44^KRGR^47^ cluster in GAG binding. A second analysis consisted of competition binding assays on heparin sepharose beads with ^125^I-CCL18 and ^125^I-^44^AAGA^47^-CCL18 performed as previously described (36) confirmed the loss of heparin binding (data not shown).

The binding capacity of the mutant was then assessed on PBLs compared to CCL18 binding (Figure 9C). These studies demonstrated that replacement of the basic residues by alanine in the 40s cluster abrogates approximately 75% of the binding capacity to PBL, suggesting either an involvement of the ^44^KRGR^47^ cluster in receptor binding, or that the majority of binding to the cells is mediated by GAGs. However by assessing the biological activity of ^44^AAGA^47^-CCL18 in chemotaxis assays no impaired cell recruitment was observed compared to CCL18 (data not shown). This indicates that the ^44^KRGR^47^ cluster mediates cell binding but not receptor-mediated signaling.

## Discussion

CCL18 has the structural features of a chemokine, being an 8-kDa basic protein, with the conserved two disulfide motif, and is proposed to have evolved as a duplication of the CCL3 gene (37). However it has several features which distinguish it from its closely related homologues, CCL3, and CCL4. Firstly, it only exists in primates, and has not been identified in any other mammalian species. Second, its receptor remains elusive after more than 15 years since its identification, despite the efforts of several laboratories. Lastly, it appears to be one of the most highly expressed chemokines, and is constitutively found at high circulating levels, and is even further upregulated in disease.

We report here that we find that it is a remarkably weak chemoattractant of leukocytes comprising PBL, with chemotactic indices rarely greater than 1.5. This could indicate either that the CCL18 receptor is uniformly expressed at low levels among these leukocyte populations or that only minor subpopulation of cells are responsive to CCL18. It could possibly be due to the chemotaxis system we are using, a bare filter assay in 96-well plates. Other laboratories using other chemotaxis methods have reported higher potencies. Boyden chamber chemotaxis experiments have been reported to induce migration with chemotactic indices of 4–6 for tonsillar B lymphocyte (13) and T lymphocytes (12), whereas transwell chambers gave chemotactic indices of approximately 3 for CD4^+^ and CD8^+^ T cells (10) and Th2 T cells (38). T cells in 96-well chambers such as we have used was reported to migrate with a chemotactic index of 2 (39). However we and others have difficulty in reproducing robust migration although we sometimes achieved a chemotactic index of 3, but sometimes also almost no migration, which we attributed to donor variation. Biological functions mediated by CCL18 have been described to be donor dependent. It was shown that CCL18 generates adaptive regulatory T cells from CD4^+^ CD25^−^ memory T cells, however this regulatory effect of CCL18 on memory CD4^+^ T cells was lost when the cells were derived from allergic subjects (40). A further study showed that CCL18 may play a role in inducing and maintaining immunological tolerance to inhaled antigens. CCL18 was shown to generate functional tolerogenic dendritic cells able to prime regulatory T cells in healthy subjects and again this effect was shown to be lost in allergic subjects (41). Thus it might be hypothesized that a desensitization phenomenon of the CCL18-receptor occurs due to the increased level of CCL18 in allergic disease. Alternatively, the low potency of CCL18 to induce chemotactic responses might also be due to a missing component required for biological activity, which might be present *in vivo*, but is lacking in *in vitro* experiments. Thus it might be possible that CCL18 mediates recruitment of these cell populations with higher potency *in vivo*.

Another possibility exists in that a more potent form is produced by post-translational modification or enzyme cleavage, for instance during inflammation. Small truncations at the N-terminus apparently did not affect activity, and moreover the full length form was shown to be predominant in the synovial fluid of RA patients. In fact we observed C-terminal truncations, which have been reported in ovarian carcinoma ascitic fluid (12), but C-terminal modifications in general do not highly impact activity. During these studies, we observed a form of CCL18 that was only seen in the synovial fluid of OA patients, and which had a mass of approximately 160 Da higher. Several post-translational modifications of chemokines have been reported. Examples are glycosylation, the most common for proteins, has been described for CCL2 (42–44), citrullination has been described for several chemokines including CXCL8 and CXCL10 (44, 45), and nitration has been reported for CCL2 (46). However none of these correspond to an additional mass of 160 Da, which could represent phosphorylation, although this has not yet been observed for chemokines. However, since we were interested in identifying more potent truncated forms, the nature of this modification and the effect on biological activity remains to be elucidated.

Besides chemotaxis, several activities for this chemokine have been reported, which could suggest that its primary role is not cell recruitment. CCL18 has been reported as a profibrotic factor, which stimulates collagen production in lung and dermal fibroblasts, whereas the effect on fibroblast proliferation was less pronounced (47). CCL18 has also been shown to be a maturation factor for monocytes inducing a pattern of cytokine expression which resembled that of IL-4-stimulated M2-spectrum macrophages (48). CCL18 has also been shown to generate adaptive regulatory T cells from CD4^+^ CD25^−^ memory T cells of healthy subjects (40). As many of the CC chemokines are known to induce costimulatory effects on T cell proliferation (49) we were interested to investigate the effect of CCL18. The investigation of the role of CCL18 in T lymphocyte proliferation revealed neither an induction of proliferation nor enhanced or reduced proliferation in response to CD3/CD28 stimulation, demonstrating no effect of CCL18 on T lymphocyte proliferation.

It should be noted that many of these activities described for CCL18 point to a role in dampening inflammation as opposed to it having a pro-inflammatory activity, which is not necessarily inconsistent with it being upregulated during many diseases. We have in fact observed properties which corroborate this anti-inflammatory role, such as antagonism of certain pro-inflammatory receptors, in addition to that of CCR3 (7) as well as its ability to displace GAG-bound chemokines (29). If this is the case, why would ticks want to inhibit CCL18? The fact that the tick *R. sanguineus* produces two CkBPs that bind CCL18 is intriguing (19). We have asked the question as to whether this binding is serendipitous since, first, it is thought to have evolved as a duplication of the CCL3 gene (37) and second, this chemokine is only expressed in primates, whereas this tick feeds on many hosts, such as dogs, rodents, cattle, deer, and humans (50). The biochemical and bioassays we have performed indicate that Evasin-1 and -4 do in fact bind and neutralize CCL18, although we did not test it as an inhibitor of chemotaxis due to the weak activity we obtain. The fact that *R. sanguieneus* encodes CCL18-binding proteins implies the relevance of this chemokine in the human host defense and one may wonder if this has not co-evolved with certain tick-borne parasitic diseases that occur in man.

Despite the fact that the chemotactic effect was not dramatic, we considered attempting to identify the elusive receptor using an expression cloning approach from the leukocyte subsets to which it migrated. We set up an assay to enable the identification of the cDNA expressing the receptor during expression cloning. Using radiolabeled CCL18 in an equilibrium competition assay to the responding subset of leukocytes, PBL, we observed similar potencies of the unlabeled chemokine and heparin in the displacement of the tracer, indicating that the binding to these cells was largely due to binding to GAGs. This was confirmed by the abrogation of the GAG binding of CCL18, since the mutant ^44^AAGA^47^-CCL18 lost the majority of its binding capacity. However the mutant was still able to induce the, albeit weak, chemotactic response, posing a conundrum as to the nature of the receptor that it activates. This issue remains to be resolved, although we hypothesize that receptor activation is attained through the remaining binding capacity to PBL demonstrated by ^44^AAGA^47^-CCL18. The substantial binding to the leukocyte cell surface must play an important role for this chemokine which merits further investigation.

In conclusion many facets of CCL18 biology remain to be elucidated, in particular its receptor. The identification of PITPNM3/Nir1 as a PTX-sensitive cell surface receptor responsible for the CCL18 induced migration of human breast cancer cells (8) indicated that this could be the receptor for this chemokine on leukocytes, but unfortunately we have shown that this seems not to be the case. Further studies will be needed to fully describe the biological relevance of this chemokine, which is the most highly expressed member of the family, and determine if its role is actually beyond that of cellular recruitment, unlike the other chemokines identified to date. Lastly the question is raised as to whether it acts on a receptor that does not belong to the classical family of 7TM GPCRs, or does it require hetero-dimerization of to date, unidentified molecules, and does its remarkable GAG-binding capacity play a role in receptor activation?

## Conflict of Interest Statement

The authors declare that the research was conducted in the absence of any commercial or financial relationships that could be construed as a potential conflict of interest.

## Author Note

Sonja Krohn performed the majority of experiments, and wrote the paper; Alexandre Garin performed Luminex analyses of the synovial fluids; Cem Gabay collected and supplied the synovial fluids, and helped in experimental design; AEIP designed the experiments, and wrote the paper.
